# Multi-Instance Multilabel Learning with Weak-Label for Predicting Protein Function in Electricigens

**DOI:** 10.1155/2015/619438

**Published:** 2015-05-05

**Authors:** Jian-Sheng Wu, Hai-Feng Hu, Shan-Cheng Yan, Li-Hua Tang

**Affiliations:** ^1^School of Geographic and Biological Information, Nanjing University of Posts and Telecommunications, Nanjing 210046, China; ^2^School of Telecommunication and Information Engineering, Nanjing University of Posts and Telecommunications, Nanjing 210046, China

## Abstract

Nature often brings several domains together to form multidomain and multifunctional proteins with a vast number of possibilities. In our previous study, we disclosed that the protein function prediction problem is naturally and inherently Multi-Instance Multilabel (MIML) learning tasks. Automated protein function prediction is typically implemented under the assumption that the functions of labeled proteins are complete; that is, there are no missing labels. In contrast, in practice just a subset of the functions of a protein are known, and whether this protein has other functions is unknown. It is evident that protein function prediction tasks suffer from *weak-label* problem; thus protein function prediction with incomplete annotation matches well with the MIML with weak-label learning framework. In this paper, we have applied the state-of-the-art MIML with weak-label learning algorithm MIMLwel for predicting protein functions in two typical real-world electricigens organisms which have been widely used in microbial fuel cells (MFCs) researches. Our experimental results validate the effectiveness of MIMLwel algorithm in predicting protein functions with incomplete annotation.

## 1. Introduction

Automated annotation of protein functions is challenging in the postgenomic era. With the rapid growth of the number of sequenced genomes, the overwhelming majority of protein products can only be annotated by computational approaches [1]. Nature usually brings multiple domains together to construct multidomain and multifunctional proteins with a vast number of possibilities [2]. The large part of genomic proteins, two-thirds in unicellular organisms and more than 80% in Metazoa, belongs to multidomain proteins [3]. In a multidomain protein, each domain can fulfill its own function independently, or in a coordinated manner with its neighbors [4]. Zhou and Zhang [5] proposed the Multi-Instance Multilabel learning (MIML) framework, where one object is represented by a bag of instances and the object is valid to have several labels simultaneously. Labels of training examples are known; however, labels of instances are unknown. We can regard each domain as an input instance and represent each biological function with an output label. In our previous study, it is disclosed that the protein function prediction problem is naturally and inherently MIML learning tasks [6]. Previously, prediction of protein functions was typically operated with the assumption that the functions of labeled proteins are complete; that is, there are no missing labels [7, 8]. Instead of things, in practice we just know a part of the functions of a protein, and whether this protein has other functions is unknown. Namely, these proteins have an incomplete annotation of their functions [9]. This kind of protein functions prediction problem with incomplete annotation can be referred to as the Multilabel Multi-Instance with weak-label learning task.

During the past several years, many Multilabel Multi-Instance learning algorithms have been developed [5, 10–12]. In our previous study, we proposed an ensemble MIML learning framework EnMIMLNN and design three algorithms for protein function prediction tasks by combining the advantage of three kinds of Hausdorff distance metrics [6]. On the other hand, in the past few years, there are multiple algorithms which have been proposed for the weak-label learning problem. Sun et al. studied the weak-label learning problem in multilabel learning and proposed a method called weak-label learning (WELL) [13]. WELL deems the fact that classification boundary for each label should go across the low density regions, and any given label will not be correlative to the majority of instances [13]. Bucak et al. [14] studied the incomplete class assignment task for annotating images and proposed an approach called MLR-GR. MLR-GR optimizes the ranking errors and group Lasso loss by a convex optimization approach. Qi et al. [15] applied the Hierarchical Dirichlet Process to append missing labels for a set of images. In addition, Wang et al. [16] designed an approach for annotating weakly labeled facial images.

Although the underlying nature of predicting protein functions with incomplete annotation matches well with the Multi-Instance Multilabel with weak-label learning framework, till now there is no attempt that has been made under this learning framework. Jiang had proposed a multilabel semisupervised learning algorithm, PfunBG, to predict protein functions, employing a birelational graph (BG) of proteins and function annotations [17]. Yu et al. [7, 8] had proposed a protein function prediction method with multilabel weak-label learning (ProWL) and a variant of ProWL (ProWLIF) in order to complete the partial annotation of proteins. Both ProWL and ProWL-IF replenish the functions of proteins under the assumption that proteins are partially annotated [7, 8]. However, multilabel learning framework is evidently degenerated versions of MIML learning framework [5, 12]. Such degenerated strategies may lose useful information in the instance spaces, and this further hurts prediction performance [5, 12]. Recently, Yang et al. [18] proposed the MIMLwel (MIML with weak-label) approach which works by assuming that highly relevant labels share some common instances, and the underlying class means of bags for each label are with a large margin. MIMLwel makes use of the label relationship, and experiments had validated the effectiveness of MIMLwel in handling the Multilabel Multi-Instance with weak-label learning problem [18].

Microbial fuel cells (MFCs) are devices that can use bacterial metabolism to produce an electrical current from a wide range of organic substrates [19]. Due to the promise of sustainable energy production from organic wastes, research has intensified in the MFCs field in the last few years [19]. In this paper, we have applied the MIMLwel algorithm for annotating protein functions in two typical real-world electricigens genomes (i.e.,* Geobacter sulfurreducens, Shewanella loihica PV-4*) which have been widely used in the MFCs researches. Our experimental results validate the effectiveness of MIMLwel algorithm in predicting functions of proteins in the electricigens genomes with incomplete annotation. In addition, it is worth mentioning that our approach is a general method for predicting protein functions with incomplete annotation.

## 2. The Formulation of the Protein Function Prediction Task with Incomplete Annotation

Nature often assembles multiple domains together to form multidomain and multifunctional proteins with high possibility, and each domain may implement its own function independently or in a cooperated manner with its neighbors. We can regard each domain as an input instance and take each biological function as an output label. Labels of the training examples are known; however, labels of instances are unknown. In our previous work, we disclose that the protein function prediction problem is naturally and inherently Multi-Instance Multilabel (MIML) learning tasks [6]. Previous studies typically predict the functions of proteins under the assumption that the functions of labeled proteins are complete; that is, there are no missing labels. In contrast, in most real cases we just know a subset of the functions of a protein, and whether this protein has other functions is unknown. Namely, these proteins have an incomplete annotation for molecular functions [9]. This type of protein function prediction problem with incomplete annotation can be inferred to as the Multilabel Multi-Instance with weak-label learning task.

We study the Multi-Instance Multilabel weak-label learning framework for protein function prediction with incomplete annotation for two tasks as illustrated in Table 1. In the tables, each row indicates the function annotation for a protein, and each column denotes a function label. Table 1(a) presents the complete annotated proteins, with 1 and 0 showing function annotations (F1–F5) on the six proteins P1–P6. In Table 1(b), 1 denotes the known relevant functions, “?” represents the missing functions and will be set to 0 s, and all the 0 s indicate the candidates for being predicted as relevant. In Task 2 as shown by Table 1(c), the definitions of 1 and 0 are the same as in Table 1(b). However, the aim of the weak-label learning is to make use of the incomplete annotated proteins (P1–P4) to predict the functions of proteins P5 and P6, which are completely unlabeled.

Formally, we represent by {*X*_*i*_, *Y*_*i*_  (*i* = 1,2,…, *m*)} the training dataset with *m* examples. *X*_*i*_ is the *i*th protein in the training dataset, and *X*_*i*_ is a bag with *n*_*i*_ instances {*x*_*i*,1_, *x*_*i*,2_,…, *x*_*i*,*n*_*i*__}. *Y*_*i*_ denotes the Gene Ontology terms which are assigned to *X*_*i*_, and *Y*_*i*_ = [*y*_*i*,1_,…, *y*_*i*,*L*_] ∈ {0,1}^*L*^ is a label vector with *L* labels, where *y*_*i*,*l*_ = +1 if the *l*th label is positive for *X*_*i*_, and 0 otherwise. Note that the labels of instances *x*_*i*,*j*_'s  (*i* = 1,…, *m*; *j* = 1,…, *n*_*i*_)  are untagged. In the MIML weak-label setting, *Y* is unknown and instead we are just given a partial label matrix $\hat{Y}\in {\left\{0,1\right\}}^{m\times L}$. Specifically, for *X*_*i*_, a label vector  $\hat{Y}=[{\hat{y}}_{i,1},\ldots ,{\hat{y}}_{i,L}]$  is given, where ${\hat{y}}_{i,l}=+1$ if the *l*th label is assigned for *X*_*i*_, and 0 otherwise. Different from the full label matrix, ${\hat{y}}_{i,l}=0$ tells us nothing. The goal is to predict all the positive labels for unseen bags [18].

## 3. Datasets and Methods

### 3.1. Data and Feature Extraction

Microbial fuel cells (MFCs) are devices that can make use of bacterial metabolism to obtain an electrical current from a wide range of organic substrates [19]. Due to the promise of sustainable energy production from organic wastes, research has booming in this field during the last few years [19]. Recently, the increased interest in MFCs technology was highlighted by the discovery of* Geobacter sulfurreducens*, a bacterial strain capable of high current production [19]. In addition, the genome-wide sequences of multiple* Shewanella* strains have been completed and annotated, opening the door to explore the diversity of their extracellular electron transfer mechanisms [20]. In this paper, two typical real-world electricigens organisms which have been widely used in microbial fuel cells (MFCs) researches (i.e.,* Geobacter sulfurreducens, Shewanella loihica PV-4*) are considered for predicting their protein functions. For each organism, complete proteome with manually annotated function has been downloaded from the Universal Protein Resource (UniProt) databank [21] (released by April, 2014) by querying the terms of {“organism name” AND “reviewed: yes” AND “keyword: Complete proteome”}.

Redundancy among protein sequences of each organism is removed by clustering operation using the* blastclust* executable program in the BLAST package [22] from NCBI with a threshold of 90% as sequence identity, and a nonredundant dataset is obtained by keeping only the longest sequence in each cluster for each organism [23]. Then, each nonredundant dataset is uploaded as a* txt* file into the Batch CD-Search servers [24] of NCBI for getting the conserved domains of each protein. For each domain, a frequency vector with 216 dimensions is employed for its representation where each element indicates the frequency of a triad type [25]. Protein function can be annotated in several ways, and the most well-known and widely used one is given by Gene Ontology Consortium [26] which offers ontology in three aspects: molecular function, biological process, and cellular location. In this study, we concentrate on the molecular function aspect. We achieve the GO molecular function terms with manual annotation for a protein from the downloaded UniProt format text file. Then, the same scheme as [27] is assigned for produce label vectors for a protein based on a hierarchal directed acyclic graph (DAG) of GO molecular function, and the latest version (December 2006) of GO function ontology is adopted as the bases of the functional terms and their relations in this work.

Under the MIML learning framework, each protein is described as a bag of instances where each instance represents a domain and is tagged with a set of GO molecular function terms (multiple labels). Detailed descriptions of the datasets, that is, complete proteome on the two above organisms, are shown in Table 2. For example, there are 373 proteins (examples) with a sum of 344 gene ontology terms (label classes) on molecular function in the* Shewanella loihica PV-4* dataset (Table 2). The average number of instances (domains) per bag (protein) is 3.14 ± 1.19, and the average number of labels (GO terms) per example (protein) is 3.55 ± 5.00 (Table 2).

### 3.2. The MIMLwel Approach

In this paper, the MIMLwel (MIML with weak-label) approach is adopted for the weak-label setting [18]. MIMLwel assumes that highly relevant labels usually share common instances, and the underlying class means of bags for each label are separated with a large margin [18].

Formally, the training dataset with *m* examples can be represented by {*X*_*i*_, *Y*_*i*_  (*i* = 1,2,…, *m*)}. *X*_*i*_ corresponds to the *i*th example in the training dataset, and *X*_*i*_ is a bag with *n*_*i*_ instances {*x*_*i*,1_, *x*_*i*,2_,…, *x*_*i*,*n*_*i*__}. *Y*_*i*_ denotes the labels which are assigned to *X*_*i*_, and *Y*_*i*_ = [*y*_*i*,1_,…, *y*_*i*,*L*_] ∈ {0,1}^*L*^ is a label vector with *L* labels, where *y*_*i*,*l*_ = +1 if the* l*th label is positive for *X*_*i*_, and 0 otherwise. Notice that the labels of instances *x*_*i*,*j*_'s (*i* = 1,…, *m*; *j* = 1,…, *n*_*i*_) are unknown. In the MIML weak-label setting, however, only a subset of labels are tagged. Specifically, for *X*_*i*_, a label vector $\hat{Y}=[{\hat{y}}_{i,1},\ldots ,{\hat{y}}_{i,L}]\in {\left\{0,1\right\}}^{m\times L}$ is given, where ${\hat{y}}_{i,l}=+1$ if the *l*th label is assigned for *X*_*i*_, and 0 otherwise. The goal is to predict all the positive labels for unseen bags [18].

For simplicity, *L* linear models were employed, and each one is for a label; that is, *f*_*l*_(*X*) = *w*_*l*_^*T*^Φ^*C*^(*X*) where each *w*_*l*_ denotes a* d*-dimensional linear predictor [*w*_*l*,1_, *w*_*l*,2_,…,*w*_*l*,*d*_]^*T*^ and *w*_*l*_^*T*^ is the transpose of *w*_*l*_. To make use of label relationship, a label relation matrix *R* ∈ [0,1]^*L*×*L*^ is considered, where $R_{l,\tilde{l}}=1$ if the two labels are related, and 0 otherwise. Let $W_{l,\tilde{l}}$ indicate $\left[w_{l},w_{\tilde{l}}\right]$ for the pair of related labels $\left(l,\tilde{l}\right)$. MIMLwel assumes that highly related labels usually share common instances, indicating that many rows of $w_{l,\tilde{l}}$ values should be equal to zero; this can be characterized by a convexly relaxed term ${\left\|w\left(l,\tilde{l}\right)\right\|}_{\left(2,1\right)}$, which is a convex relaxation of ${\left\|w\left(l,\tilde{l}\right)\right\|}_{\left(2,0\right)}$. Thus, the goal of MIMLwel is to obtain *W* = [*w*_1_,…, *w*_*L*_] and an output matrix $\hat{Y}$ to meet that

(1) min⁡W,Y− ⁡−η∑l=1LVy−i,l,Xii=1m,wl+∑1<l, l~≤LRl,l~Wl,l~2,12s.t. hhY−l−Y^l1Y^l1≤ϵ;y−i,l=y^i,l if  y^i,l=1,  ∀l=1,…,L,

where *V* is a loss function for each label, |·|_1_ represents the *l*_1_-norm, *ϵ* controls the sparsity of ${\left|{\overset{-}{Y}}_{l}-{\hat{Y}}_{l}\right|}_{1}$, and *η* trades off the empirical risk and model complexity.

### 3.3. Experimental Configuration

In this paper, we adopt three popular multilabel learning evaluation criteria, that is,* Hamming loss (HL), macro-F1 (maF1), and micro-F1 (miF1)* [28–30].* Hamming loss* assesses how many times on average a bag label pair is wrongly predicted. The smaller the value of hamming loss, the better the performance.* Macro-F1* computes* F1* measure on each class label at first and then averages over all class labels.* Macro-F1* is more influenced by the performance of the classes owning fewer examples. The larger the value of* macro-F1*, the better the performance.* Micro-F1* globally calculates the* F1* measure on the predictors over all bags and all class labels.* Micro-F1* is more affected by the performance of the classes involving more examples. The larger the value of* micro-F1*, the better the performance. The definition of these criteria can be found in [30]. We repeat 10-fold cross validation for each dataset ten times and the mean ± std. performances are presented for the proposed and compared methods.

## 4. Results and Discussion

### 4.1. Performance of the MIMLwel Method

In our experiments we consider four weak-label ratios (W.L.R.) [18], defined as ${\left|{\hat{Y}}_{\cdot ,l}\right|}_{1}/{\left|Y_{\cdot ,l}\right|}_{1}$, from 20% to 80% with 20% as the interval.  Table 3 illustrates the performances of MIMLwel based on each kind of W.L.R. on the* Geobacter sulfurreducens* and* Shewanella loihica PV-4* datasets. For each evaluation criterion, ↑(↓) indicates the larger (smaller), the better the performance; the best results on each evaluation criterion are highlighted in boldface. As indicated in Table 3, the results show that, with the rising of W.L.R., the model performance of MIMLwel has been greatly improved.

**Figure 1:**
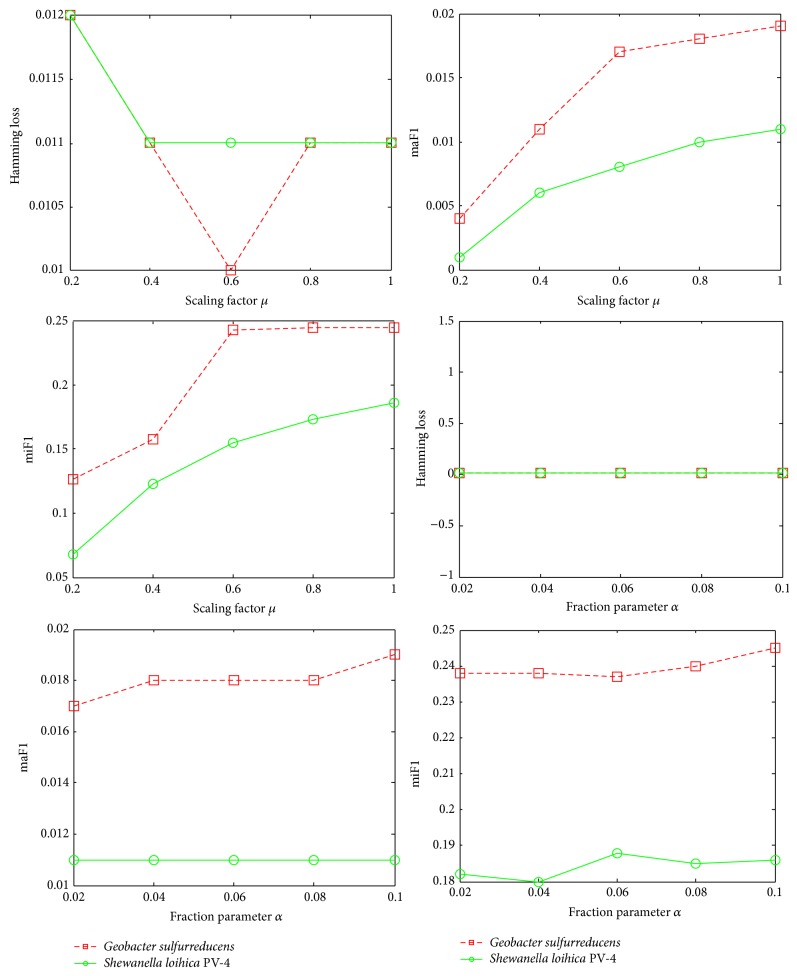
The performance of MIMLwel on all two datasets with 80% weak-label ratios (W.L.R.) under different values of scaling factor*μ* when the fraction parameter*α* is fixed to 0.1 and different values of the fraction parameter*α* when the scaling factor*μ* is fixed to 1.0. The performance of MIMLwel reaches the perk in most cases by setting the scaling factor*μ* to 1.0 and the fraction parameter *α* to 0.1.

The MIMLwel approach [18] involves two different parameters, that is, the scaling factor  *μ* and the fraction parameter  *α*.  Figure 1 shows how the MIMLwel algorithm is implemented on the two datasets with 80% weak-label ratios (W.L.R.) under different parameter configurations, where the performance is measured in terms of* HL, maF1, *and* miF1*. Here,  *μ* varies from 0.2 to 1.0 with an interval of 0.2 when  *α* is fixed to 0.1, and  *α* increases from 0.02 to 0.1 with an interval of 0.02 with the fixed  *μ* equal to 1.0. It is indicated that the performance of the MIMLwel algorithms achieves the perk in most cases by setting the scaling factor  *μ* to 1.0 and the fraction parameter  *α* to 0.1. In this paper, the MIMLwel algorithm is implemented by setting the scaling factor  *μ* to 1.0 and the fraction parameter  *α* to 0.1.

### 4.2. Performance Comparison

In this paper, we compare the MIMLwel algorithm with four state-of-the-art MIML algorithms, that is, MIMLkNN [31], MIMLNN [12], MIMLRBF [32], and MIMLSVM [5], under different configuration of weak-label ratios (W.L.R.) on the* Geobacter sulfurreducens* dataset (Table 4) and* Shewanella loihica PV-4 *dataset (Table 5). The codes of compared MIML algorithms are shared by their authors, and these algorithms are implemented using the best parameters reported in the papers. Specifically, for MIMLkNN, the number of nearest neighbors and the number of citers are set to 10 and 20, respectively [31]; for MIMLNN, the number of clusters is set to 40% of the training bags, and the regularization parameter used to compute matrix inverse is set to 1 [12]; for MIMLRBF, the scaling factor and the fraction parameter are set to 0.6 and 0.1, respectively [32]; for MIMLSVM, the number of clusters is set to 20% of the training bags and the Gaussian kernel width is set to 0.2 [5]. Tables 4 and 5 summarize the experimental results of each compared algorithm on the* Geobacter sulfurreducens* dataset and* Shewanella loihica PV-4* dataset, respectively. For each evaluation criterion, “↓” indicates “the smaller the better,” while “↑” indicates “the bigger the better.” Furthermore, the best results on each evaluation criterion are highlighted in boldface. It is indicated that the MIMLwel algorithm performs quite well in terms of most criteria in two datasets (Tables 5 and 6). Specifically, paired* t*-tests at 95% significance level indicate that the MIMLwel algorithm achieves significantly better performance than compared methods in most cases, as shown by the overwhelming ●'s in Tables 4 and 5.

### 4.3. Case Study

Table 6 presents two example results. The first protein with the UniProt ID “Q74BW7” from the* Geobacter sulfurreducens* organism has seven ground-truth labels: {GO:0008270, GO:0046872, GO:0000287, GO:0051539, GO:0030145, GO:0005506, GO:0004160}. After training examples with 80% weak-label ratios by different MIML methods, the trained model is then used to predict the GO molecular function labels of this protein. The correctly predicted GO molecular function labels by each method are highlighted in boldface. It is shown in Table 6 that MIMLwel successfully predicts most of the ground-truth labels (6/7); however, it predicts one more label, that is, GO:0005524, which is not in the ground-truth list. Nevertheless, the label GO:0005524 that denotes “ATP binding” may be not a conflict with the true molecular function in UniProt. MIMLRBF and EnMIMLNN{metric} predict two ground-truth labels but still miss a lot (5/7). MIMLNN reports no prediction result, and MIMLSVM only reports a wrong GO molecular function label. Similar situation also happen in the second example with the UniProt ID “A3QFX5” from the* Shewanella loihica PV-4* organism as indicated in Table 6.

## 5. Conclusion

In our previous study, we disclosed that the protein function prediction problem is naturally and inherently Multi-Instance Multilabel (MIML) learning tasks. Automated protein function prediction was typically implemented under the assumption that the functions of labeled proteins are complete; that is, there are no missing labels. In contrast, in practice just a subset of the functions of a protein are known, and whether this protein has additional functions is unknown. It is evident that the protein function prediction tasks suffer from weak-label problems, and we disclose that prediction of protein functions with incomplete annotation matches well with the MIML with weak-label learning framework in this paper. In this paper, we have applied the state-of-the-art MIML with weak-label learning algorithm MIMLwel for predicting protein function in two typical real-world electricigens organisms which have been widely used in microbial fuel cells (MFCs) researches. Our experimental results show that MIMLwel is superior to most state-of-the-art MIML algorithms, which validates the effectiveness of MIMLwel algorithm in predicting protein functions with incomplete annotation.

## Figures and Tables

**Table:** (a) Original

	F1	F2	F3	F4	F5
P1	0	1	0	1	0
P2	0	0	1	0	1
P3	1	1	0	0	1
P4	0	1	1	0	0
P5	1	0	0	1	0
P6	0	1	0	0	0

**Table:** (b) Task 1

	F1	F2	F3	F4	F5
P1	0	?	0	1	0
P2	0	0	?	?	1
P3	1	?	0	?	1
P4	?	1	1	0	0
P5	1	0	?	?	0
P6	0	1	?	0	0

**Table:** (c) Task 2

	F1	F2	F3	F4	F5
P1	0	?	0	1	0
P2	0	0	?	?	1
P3	1	?	0	?	1
P4	?	1	1	0	0
P5	?	?	?	?	?
P6	?	?	?	?	?

**Table 2:** Characteristics of the data sets.

Organism	Examples	Classes	Instances per bag (mean ± std.)	Labels per example (mean ± std.)
*Geobacter sulfurreducens *	379	320	3.20 ± 1.21	3.14 ± 3.33
*Shewanella loihica PV-4 *	373	344	3.14 ± 1.19	3.55 ± 5.00

**Table 3:** Performance of the MIMLwel methods with different weak-label ratios on two datasets.

Datasets	W.L.R.	HL↓	maF1↑	miF1↑
*Geobacter sulfurreducens *	20%	0.010 ± 0.002	0.003 ± 0.004	0.032 ± 0.035
	40%	0.010 ± 0.002	0.009 ± 0.005	0.116 ± 0.038
	60%	0.010 ± 0.002	0.016 ± 0.006	0.201 ± 0.034
	80%	0.011 ± 0.001	**0.019 ± 0.007**	**0.245 ± 0.050**

*Shewanella loihica PV-4 *	20%	0.013 ± 0.002	0.009 ± 0.008	0.145 ± 0.111
	40%	0.010 ± 0.002	0.005 ± 0.003	0.092 ± 0.039
	60%	0.011 ± 0.003	0.010 ± 0.006	0.167 ± 0.072
	80%	0.011 ± 0.003	**0.011 ± 0.005**	**0.186 ± 0.043**

**Table 4:** Comparison results (mean ± std.) of MIMLwel models with four state-of-the-art MIML methods with different weak-label ratios on the *Geobacter sulfurreducens* dataset.

W.L.R.	Methods	HL↓	maF1↑	miF1↑
20%	MIMLwel	**0.010 ± 0.002**	0.003 ± 0.004	**0.032 ± 0.035**
	MIMLNN	**0.010 ± 0.002**	0.000 ± 0.000	0.000 ± 0.000 ●
	MIMLRBF	**0.010 ± 0.002**	0.002 ± 0.003	0.002 ± 0.003 ●
	MIMLSVM	0.012 ± 0.002	**0.005 ± 0.003**	0.005 ± 0.003 ●
	EnMIMLNN {metric}	**0.010 ± 0.002**	0.002 ± 0.002	0.001 ± 0.002 ●

40%	MIMLwel	**0.010 ± 0.002**	**0.009 ± 0.005**	**0.116 ± 0.038**
	MIMLNN	**0.010 ± 0.002**	0.000 ± 0.000	0.000 ± 0.000 ●
	MIMLRBF	**0.010 ± 0.002**	0.004 ± 0.004	0.003 ± 0.003 ●
	MIMLSVM	0.012 ± 0.001	0.006 ± 0.003	0.006 ± 0.003 ●
	EnMIMLNN {metric}	**0.010 ± 0.002**	0.003 ± 0.004	0.003 ± 0.003 ●

60%	MIMLwel	0.010 ± 0.002	0.**016 ± 0.006**	**0.201 ± 0.034**
	MIMLNN	0.010 ± 0.001	0.001 ± 0.001	0.001 ± 0.001 ●
	MIMLRBF	**0.009 ± 0.001**	0.009 ± 0.007	0.008 ± 0.007 ●
	MIMLSVM	0.011 ± 0.001	0.008 ± 0.003	0.008 ± 0.003 ●
	EnMIMLNN {metric}	0.010 ± 0.001	0.009 ± 0.004	0.008 ± 0.004 ●

80%	MIMLwel	0.011 ± 0.001	**0.019 ± 0.007**	**0.245 ± 0.050**
	MIMLNN	0.010 ± 0.001	0.002 ± 0.001 ●	0.002 ± 0.001 ●
	MIMLRBF	**0.009 ± 0.000**	0.009 ± 0.004 ●	0.008 ± 0.004 ●
	MIMLSVM	0.011 ± 0.001	0.008 ± 0.002 ●	0.008 ± 0.002 ●
	EnMIMLNN {metric}	**0.009 ± 0.001**	0.013 ± 0.004	0.012 ± 0.004 ●

**Table 5:** Comparison results (mean ± std.) of MIMLwel models with four state-of-the-art MIML methods with different weak-label ratios on the *Shewanella loihica PV-4* dataset.

W.L.R.	Methods	HL↓	maF1↑	miF1↑
20%	MIMLwel	0.013 ± 0.002	**0.009 ± 0.008**	**0.145 ± 0.111**
	MIMLNN	**0.010 ± 0.002**	0.000 ± 0.000	0.000 ± 0.000 ●
	MIMLRBF	0.011 ± 0.003	0.001 ± 0.001	0.001 ± 0.001 ●
	MIMLSVM	0.012 ± 0.002	0.005 ± 0.002	0.004 ± 0.002 ●
	EnMIMLNN {metric}	**0.010 ± 0.003**	0.001 ± 0.001	0.001 ± 0.001 ●

40%	MIMLwel	**0.010 ± 0.002**	**0.005 ± 0.003**	**0.092 ± 0.039**
	MIMLNN	**0.010 ± 0.002**	0.000 ± 0.000	0.000 ± 0.000 ●
	MIMLRBF	**0.010 ± 0.002**	0.001 ± 0.002	0.001 ± 0.002 ●
	MIMLSVM	0.012 ± 0.002	0.004 ± 0.002	0.004 ± 0.002 ●
	EnMIMLNN {metric}	**0.010 ± 0.002**	0.001 ± 0.003	0.001 ± 0.003 ●

60%	MIMLwel	0.011 ± 0.003	**0.010 ± 0.006**	**0.167 ± 0.072**
	MIMLNN	**0.010 ± 0.003**	0.001 ± 0.001	0.001 ± 0.001 ●
	MIMLRBF	**0.010 ± 0.004**	0.004 ± 0.004	0.003 ± 0.003 ●
	MIMLSVM	0.012 ± 0.003	0.005 ± 0.001	0.005 ± 0.002 ●
	EnMIMLNN {metric}	**0.010 ± 0.003**	0.005 ± 0.003	0.004 ± 0.003 ●

80%	MIMLwel	0.011 ± 0.003	**0.011 ± 0.005**	**0.186 ± 0.043**
	MIMLNN	0.010 ± 0.003	0.002 ± 0.001	0.001 ± 0.001 ●
	MIMLRBF	**0.009 ± 0.003**	0.008 ± 0.005	0.007 ± 0.005 ●
	MIMLSVM	0.012 ± 0.003	0.005 ± 0.002	0.005 ± 0.001 ●
	EnMIMLNN {metric}	0.010 ± 0.003	0.006 ± 0.004	0.005 ± 0.003 ●

**Table 6:** Comparison results on two examples.

Organism/UniProt ID	Molecular function in UniProt	Methods	GO molecular function list		
*Geobacter sulfurreducens*/ Q74BW7	(1) 4 iron, 4 sulfur cluster binding (2) Dihydroxy-acid dehydratase activity (3) Metal ion binding	Ground truth	GO:0008270	GO:0046872	GO:0000287
			GO:0051539	GO:0030145	GO:0005506
			GO:0004160		
		MIMLwel	GO:0005524	**GO:0008270**	**GO:0046872 **
			**GO:0000287**	**GO:0030145**	**GO:0005506**
		MIMLNN	Null		
		MIMLRBF	**GO:0000287**	**GO:0005506**	
		MIMLSVM	GO:0050567		
		EnMIMLNN {metric}	**GO:0000287**	**GO:0005506**	

*Shewanella loihica PV-4*/A3QFX5	(1) ATP binding (2) Nucleoside-triphosphatase activity (3) Zinc ion binding	Ground truth	GO:0003924	GO:0005524	GO:0004386
			GO:0008270	GO:0016887	GO:0046961
			GO:0005215	GO:0017111	GO:0004004
			GO:0008094	GO:0008565	
		MIMLwel	**GO:0005524**	**GO:0004386**	**GO:0016887 **
			**GO:0046961**	**GO:0004004**	**GO:0008094**
			GO:0043565		
		MIMLNN	Null		
		MIMLRBF	**GO:0005524**	**GO:0004386**	**GO:0016887 **
			**GO:0046961**	**GO:0004004**	**GO:0008094**
		MIMLSVM	**GO:0008270**		
		EnMIMLNN {metric}	**GO:0005524**	**GO:0016887**	**GO:0004004**
